# Surface Topography Prediction Model in Milling of Thin-Walled Parts Considering Machining Deformation

**DOI:** 10.3390/ma14247679

**Published:** 2021-12-13

**Authors:** Zhitao Chen, Caixu Yue, Xianli Liu, Steven Y. Liang, Xudong Wei, Yanjie Du

**Affiliations:** 1 Key Laboratory of Advanced Manufacturing and Intelligent Technology, Ministry of Education, Harbin University of Science and Technology, Harbin 150080, China; zhitao19@163.com (Z.C.); xianli.liu@hrbust.edu.cn (X.L.); weixudong686@163.com (X.W.); yanjie.work@outlook.com (Y.D.); 2 Georgia Institute of Technology, George W. Woodruff School of Mechanical Engineering, Atlanta, GA 30332, USA; steven.liang@me.gatech.edu

**Keywords:** thin-walled parts, surface topography, chip thickness, machining deformation

## Abstract

With the continuous improvement of the performance of modern aerospace aircraft, the overall strength and lightweight control of aircraft has become a significant feature of modern aerospace parts. With the wide application of thin-walled parts, the requirements for dimensional accuracy and surface quality of workpieces are increasing. In this paper, a numerical model for predicting surface topography of thin-walled parts after elastic deformation is proposed. In view of the geometric characteristics in the cutting process, the cutting force model of thin-walled parts is established, and the meshing relationship between the tool and the workpiece is studied. In addition, the influence of workpiece deformation is considered based on the beam deformation model. Cutting force is calculated based on deformed cutting thickness, and the next cutting–meshing relationship is predicted. The model combines the radial deflection of the workpiece in the feed direction and the changing meshing relationship of the tool–workpiece to determine the three-dimensional topography of the workpiece. The error range between the experimental and the simulation results of surface roughness is 7.45–13.09%, so the simulation three-dimensional morphology has good similarity. The surface topography prediction model provides a fast solution for surface quality control in the thin-walled parts’ milling process.

## 1. Introduction

Aeronautical structural components are widely used in the new generation of aerospace vehicles due to lightweight, high specific strength and compact space structure. There are higher requirements for the efficient and high-precision manufacturing of aviation structural parts because of the improvement of aircraft performance [1].

In order to ensure the parts’ quality and improve the machining efficiency, it is necessary to carry out the simulation research of milling thin-walled parts, including the prediction of milling force, size error, machining deformation and surface quality. Surface quality (surface roughness) is one of the important indexes that affect the workpiece quality, which is related to the wear resistance, fatigue strength and assembly relationship among components [2]. With the improvement of computers’ computing ability, the research cost of numerical simulation to predict surface roughness is lower, the efficiency is higher and the phenomenon of poor economy of experimental methods will not occur. Predicting the surface profiles of the workpiece surface in the cutting process is the key to improve surface micro-topography. By analyzing the influencing factors of surface topography, the surface-forming mechanism, milling force and workpiece deformation are discussed, and the meshing mathematical model of the tool–workpiece is established. Through the simulation of surface topography, the predicted surface roughness is obtained. Compared with the experimental results, the simulation method has a high performance price ratio.

Prediction and simulation of macroscopic milling surface topography is an active research topic of many scholars. Based on the cutting parameters and tool geometry, a general surface topography model is established. The model is improved by considering the factors such as tool run-out, tool deflection, dynamic system and tool wear. Lee [3] introduced an acceleration signal to establish a simulation model of the machined surface in high-speed milling, which solved the problem that the influence of spindle vibration on surface morphology was not considered in previous models. The cutting vibration is converted into a polynomial to solve the discrete position coordinates in the feed direction, and the prediction model of surface roughness is established [4,5]. Yang [6] used variable-pitch end mills to predict the surface topography. In the prediction model of surface topography considering plastic deformation in the side milling process, through experiments and sensitivity analysis, it was found that tool deflection is the key factor affecting roughness. The analytical models of surface morphology prediction were established by analyzing tool vibration, calculating the geometric structure of face end mills’ blade and axial radial run-out. Arizmendi [7] established a surface morphology prediction model considering tool geometry and installation error. Since this model does not consider the influence of cutting force on surface texture, the best application effect is face milling. The surface topography of peripheral and face milling was modeled by numerical simulation, and the simulation results are in good agreement with the experimental results [8,9,10]. The machining time is discretized in the dynamic model, and the three-dimensional matrix of the discrete points on the cutting edge at different times in the cutting process is recorded. The geometry of the machined surface in the dynamic cutting process is constructed through iterative calculation [10]. Yan [11] introduced tool vibration into milling dynamics, studied the influence of process parameters on surface roughness and obtained the result that the feed per tooth had the greatest influence on surface roughness during side milling, which provided a basis for parameter optimization of surface roughness. Maruda [12] evaluated the surface integrity of dry cutting and different lubrication cutting. Then, through the experimental surface morphology parameters and hardening results, the MQCL + EP/AW cooling method was selected to obtain uniform distribution of the valley value and peak value. Zhang [13] established the prediction model of surface roughness by considering the spindle speed and cutting parameters, and studied the spectrum with the 3D surface roughness figure. The Gaussian regression model was used to predict surface roughness of different machining parameters, and the accuracy rate was 84.3%. Chen [14] established the dynamic cutting forces model by considering the regeneration effect of tool run-out and axial drift, and proposed the surface topography simulation method based on the Z-map model. According to the zero-order analysis method, the stable cutting lobe diagram was obtained, and the surface roughness value near the resonance spindle speed and the left side of the lobe diagram was better. When predicting the surface topography in the dynamic milling system, relevant modal experiments were needed, which increased the computational cost of surface topography prediction. Pimenov [15] analyzed the influence of a face milling cutter cutting on the surface of the workpiece and the variation of the cutting component when in the relative position of the face milling cutter and the workpiece. The surface roughness was obtained by analyzing the tool–workpiece position relationship, milling dynamics and spindle speed, which provided a practical scheme for actual machining. Wojciechowski [16] studied the surface roughness of the ball-end-mill with different values of tool overhang. It was found that the surface roughness of a small value of tool overhang was related to tool motion geometry and machining system error, and the surface of a large value of tool overhang was related to cutting force and tool dynamic deflection.

With the wide application of thin-walled parts in the field of modern aircraft, the research on the surface topography of thin-walled parts has entered a new field. Due to the low stiffness in the machining process of thin-walled parts, improving the quality of parts (geometric precision, surface quality and assembly accuracy) has become a challenging problem [17]. Experimental methods [18], analytical methods based on cutting mechanism, tool differential geometry theory and the workpiece geometric structure analysis method [19,20] in the process of surface morphology prediction were widely used. In the 1980s, the prediction model of surface topography for thin-walled parts was established [21], which was continuously improved and updated. The surface topography is predicted by considering tool geometric tolerance, tool geometric accuracy and feed in the cutting process [22]. The local stiffness change of thin-walled parts can maintain the material removal rate and stability of cutting force by changing the feed [23]. Xie [24] analyzed surface structure and tool path characteristics of the workpiece, and considered the constraints of acceleration and impact. The smoothness model of thin-walled parts was derived, the contour error was obtained and the surface roughness was evaluated.

When the dynamic deformation of thin-walled parts is obtained in advance, the milling force is simplified as the input value. The vibration equation is solved by the modal superposition method, the dynamic deformation equation and cutting trajectory coordinates under the excitation of milling force are obtained and the surface roughness is evaluated [2]. The tool geometry parameters are also prominent problems for milling vibration and surface topography of thin-walled parts. Cutting simulations and experimental studies are carried out on various tool geometric structures. Tool design and process parameters’ optimization for thin-walled parts’ milling are of great significance [25]. Wang [26] calculates the local coordinates of the instantaneous tool, and obtains the tool point cloud by matrix transformation. The 3D surface topography is obtained according to the Boolean operation among small envelope bodies of the contour component and small boundaries. Zhang [27] established an accurate model of the instantaneous cutting thickness in the 5-axis side milling process, which was calculated through the linear iterative process and provided an effective process method for improving machining accuracy and good surface quality. Many new models for predicting surface morphology have been established, but many methods have high computational costs and require the assistance of finite element software. In the range of reasonable error, the numerical model with lower calculation costs and faster calculation speed becomes a good solution.

In the establishment of a surface topography model, the above analysis method assumes that the material removal area is a fixed value, and there is no comprehensive analysis of the coupling relationship between force and deformation. In order to improve the surface quality, many scholars have also studied the optimization of process parameters. By changing spindle speed, feed, cutting depth and cutting method, the influence of process parameters on surface topography is studied [28]. The adjustment and optimization of milling parameters are more convenient in practical operation with the goal of efficient removal of materials and good surface quality [29]. Therefore, intelligent algorithms are introduced into the machining process. The genetic algorithm, particle swarm optimization and other algorithms are used to optimize the process parameters to obtain the ideal goal [30,31]. Yildiz [32] developed the cuckoo search algorithm (CS) for processing parameter optimization. By comparing the ant colony algorithm, immune algorithm, hybrid particle swarm algorithm, etc., the effectiveness and stability of CS were proven. However, intelligent algorithms require a large number of datasets for training, which increases the computational cost of model prediction. At the same time, the actual processing conditions are different, the factors affecting the surface quality will change and the prediction results will also be biased. However, the factors affecting the surface topography are in a closed loop, so it is necessary to analyze the coupling relationship of related factors.

In the actual processing of thin-walled parts, in order to ensure the processing efficiency, the tools with good stiffness and large diameter are selected. The deformation is concentrated on the workpiece, which changes the tool–workpiece contact relationship and affects the accuracy of the surface topography. Therefore, an effective and fast surface topography prediction model can provide theoretical support for improving the processing quality.

In order to improve the prediction accuracy of surface topography in the milling process for thin-walled parts, this paper proposes a surface topography prediction model considering the elastic deformation of thin-walled parts and the tool–workpiece contact relationship. Through the basic cutting force model and characteristics of the cutting contact relationship, the coupling expression of elastic deformation and milling force of thin-walled parts is established. The instantaneous deformation matrix is obtained by the iterative calculation method, and the boundary of the forming zone on the side milling surface of thin-walled parts after deformation is redefined. The surface topography is predicted by using the surface formation mechanism, instantaneous tool–workpiece contact model and instantaneous workpiece deformation. The model is verified by comparing the simulation prediction results with experimental results.

## 2. Force and Deformation Model

### 2.1. Basic Mechanical Model

**Figure 1:**
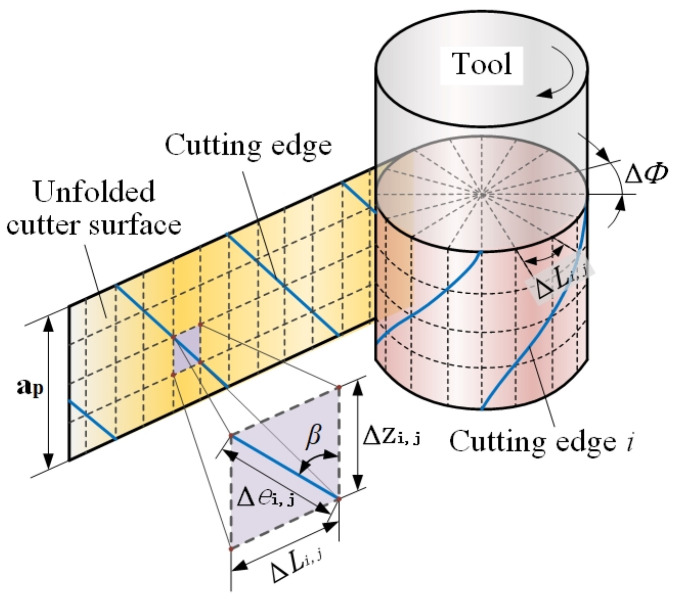
Discretization of an end milling tool.

The process and tool geometry parameters have an effect on the workpiece surface profile at the intersection of each cutting edge and machined surface [29]. Accurate expression of the tool geometric mathematical model and cutting parameters is a prerequisite for predicting the cutting force and deformation. Therefore, in the calculation process, the milling tool is discretized along the axis direction, the axial cutting depth ap direction is divided into *j* disks and each disk height is Δz, as illustrated in Figure 1.

The milling tool cylinder is discretized into *m* elements in the radial direction, and $\Delta L_{i,j}$ is the arc length of each unit, as follows:

(1) $$\Delta L_{i,j}=\frac{\pi D}{m}$$

where *D* is the tool diameter, and m is the number of segmentations. The relationship among Δz, ΔL and the helix angle β is as follows:

(2) $$\Delta z_{i,j}=\frac{\Delta L_{i,j}}{\tan\beta }$$

The relationship among the length of a tool flute, Δe, of a single disk, Δz and β is as follows:

(3) $$\Delta e_{i,j}=\frac{\Delta z_{i,j}}{\cos\beta }$$

Due to the rapid change of the tool position in the cutting process, the trajectory of the cutting edge is a three-dimensional space curve. The discretization of the end milling tool is necessary in the prediction of surface topography.

An effective cutting force calculation model is the basis of predicting the deformation and surface topography thin-walled parts. Altintas and Budak established the mechanical cutting force model with good adaptability and high calculation accuracy [33,34], which well-describes the cutting physical process in the shear zone. This model is more versatile and can be applied to face milling and side milling processes. The oblique cutting process of the helical end milling tool is described as three vectors, of shear force component, Ft, radial force component, Fr, and axial force component, Fa. In the model, the shear force coefficients *K_tc_*, *K_rc_* and *K_ac_* and the edge force coefficients *K_te_*, *K_re_* and *K_ae_* are defined according to the material properties. The tangential, radial and axial forces of the *j*th disk with the *i*th edge are expressed as follows:

(4) $$\left[\begin{array}{c} dF_{t,i,j}\\ dF_{r,i,j}\\ dF_{a,i,j} \end{array}\right]=g\left(\phi \right)\left(\left[\begin{array}{c} K_{tc,i,j}\\ K_{rc,i,j}\\ K_{ac,i,j} \end{array}\right]h_{i,j}\left(\phi \right)dz_{i,j}+\left[\begin{array}{c} K_{te,i,j}\\ K_{re,i,j}\\ K_{ae,i,j} \end{array}\right]dz_{i,j}\right)$$

(5) $$g(\phi )=\{\begin{array}{ll} 1, & \mathrm{if}\phi_{st}\leq \phi_{i,j}\mathrm{mod}2\pi \leq \phi_{ex}\\ 0, & \mathrm{otherwise}. \end{array}$$

where g(ϕ) is a step function representing the cutting state of the current cutting disk, and $\phi_{st}$ and $\phi_{ex}$ are the entry angle and exit angle, respectively.

The cutting force coefficient is obtained by the cutting database [35], and its calculation formula is:

(6) $$\begin{array}{l} K_{tc,i,j}=\frac{\tau_{s}}{\sin\psi_{n}}\frac{\cos(\beta_{n}-\alpha_{n})+\tan\beta \tan\eta \sin\beta_{n}}{\sqrt{\cos^{2}(\psi_{n}+\beta_{n}-\alpha_{n})+\tan^{2}\eta \sin^{2}\beta_{n}}}\\ K_{rc,i,j}=\frac{\tau_{s}}{\sin\psi_{n}\cos\beta }\frac{\sin(\beta_{n}-\alpha_{n})}{\sqrt{\cos^{2}(\psi_{n}+\beta_{n}-\alpha_{n})+\tan^{2}\eta \sin^{2}\beta_{n}}}\\ K_{ac,i,j}=\frac{\tau_{s}}{\sin\psi_{n}}\frac{\cos(\beta_{n}-\alpha_{n})\tan\beta -\tan\eta \sin\beta_{n}}{\sqrt{\cos^{2}(\psi_{n}+\beta_{n}-\alpha_{n})+\tan^{2}\eta \sin^{2}\beta_{n}}} \end{array}$$

where $\tau_{s}$ is the shear stress at the shear plane, and $\psi_{n}$ and $\beta_{n}$ are the normal shear and friction angles, respectively.

Convert the tangential force, $dF_{t,i,j}$, radial force, $dF_{r,i,j}$, and axial force, $dF_{a,i,j}$, to the cutting force in the *X*, *Y* and *Z* directions, as:

(7) $${\left[F_{X,i,j},F_{Y,i,j},F_{Z,i,j}\right]}^{T}=T(\phi ){\left[F_{t,i,j},F_{r,i,j},F_{a,i,j}\right]}^{T}$$

(8) $$T(\phi )=\left[\begin{array}{ccc} \sin(\phi ) & -\cos(\phi ) & 0\\ \cos(\phi ) & \sin(\phi ) & 0\\ 0 & 0 & 1 \end{array}\right]$$

The total cutting force for all disks is calculated as:

(9) $$F_{X}(\phi )=\sum_{i,j}F_{X,i,j}(\phi ),F_{Y}(\phi )=\sum_{i,j}F_{Y,i,j}(\phi ),F_{Z}(\phi )=\sum_{i,j}F_{Z,i,j}(\phi )$$

### 2.2. Beam Deflection Model

Due to the weak rigidity of the thin-walled parts, the cutting force causes the workpiece to deform. The purpose of simplifying the thin plate into the beam model is to improve calculation accuracy and efficiency. In the calculation of workpiece deformation, only the deformation generated by the cutting area is calculated, which will not affect the properties of other positions of the workpiece. The geometric shape of thin-walled parts changes significantly during the machining process, and the cross-section presents a ladder-type contour. In the analysis, the workpiece is regarded as a fixed free beam, and the free end presents a ladder contour to describe the geometric changes of the workpiece in the material removal process.

**Figure 2:**
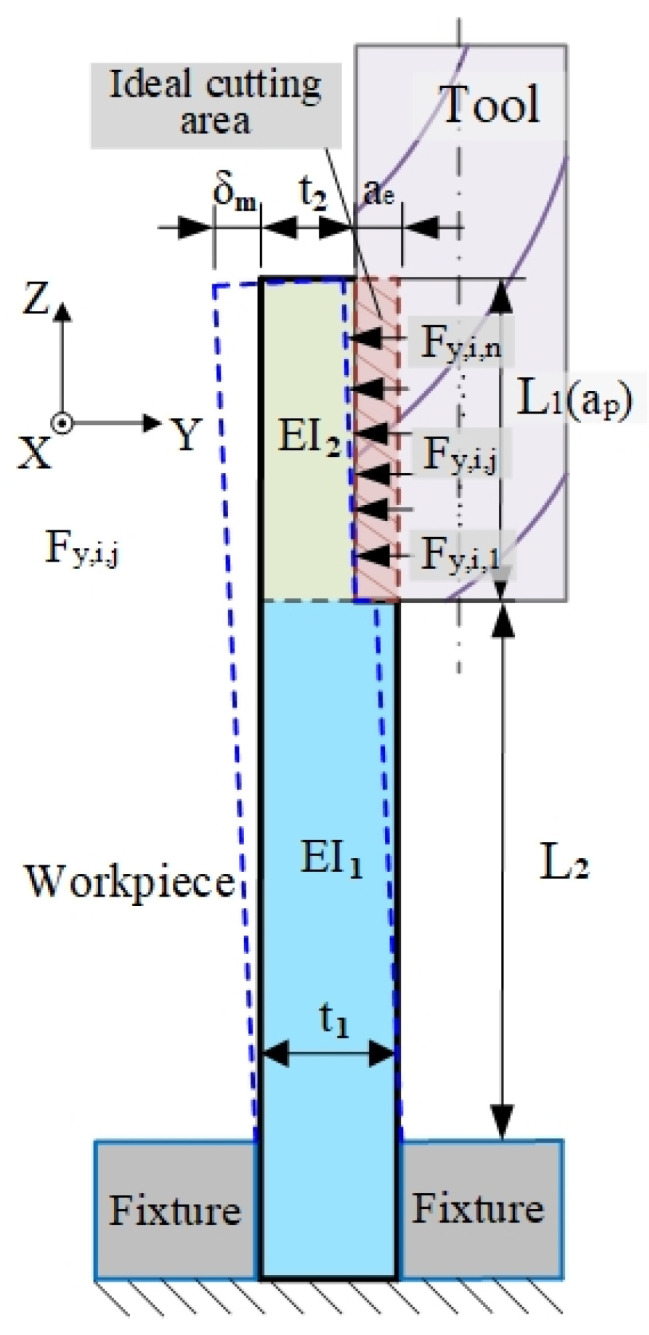
Beam model of the workpiece [37].

Figure 2 shows the cantilever beam geometric structure of the thin-walled parts with one end fixed in the milling process. As the cutting process continues, the cutting area is removed from the workpiece and the machined surface profile is gradually formed until the ideal geometric shape is obtained. The deformation caused by the cutting force at the free end is calculated by the Euler–Bernoulli theory [36].

The deformation at the free end is expressed as:

(10) $$\delta_{m}\left(X\right)=\delta_{L1}^{}\left(X_{1}\right)+\delta_{L2}\left(X_{2}\right)$$

where $\delta_{L1}^{}\left(X_{1}\right)$ is the unprocessed deflection variable and $\delta_{L2}\left(X_{2}\right)$ is the processed deflection variable.

The formula of beam deflection is expressed by the piecewise function:

(11) $$\delta_{m}\left(X\right)=\{\begin{array}{cc} \frac{FX_{}^{3}}{6EI_{1}}-\frac{FL_{1}X_{}^{2}}{2EI_{1}}-\frac{FL_{2}X_{}^{2}}{2EI_{1}} & 0<X\leq L_{1}\\ \delta_{L1}\left(L_{1}\right)+\frac{F{\left(X-L_{1}\right)}^{3}}{6EI_{1}}-\frac{FL_{2}{\left(X-L_{1}\right)}_{}^{2}}{2EI_{1}}+\theta \left(L_{1}\right)\left(X-L_{1}\right) & L_{1}<X\leq L_{1}-L_{2} \end{array}$$

### 2.3. Instantaneous Cutting Thickness Model

**Figure 3:**
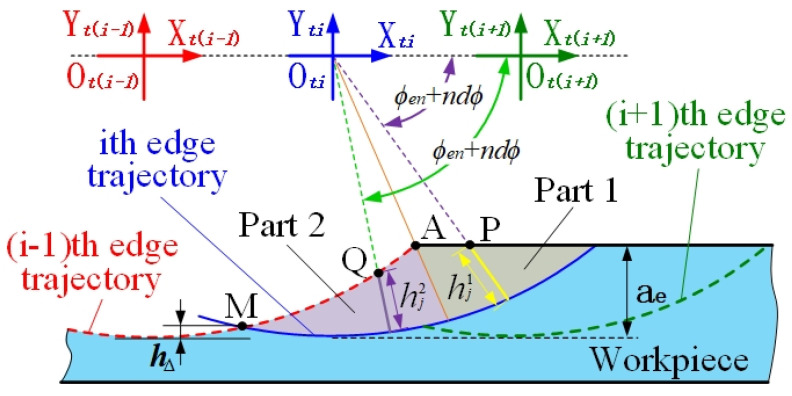
2D cutting thickness model.

During the milling of thin-walled parts, the machining deformation changes the tool–workpiece contact relationship. Instantaneous cutting thickness, $h_{i,j}$, is changed by tool instantaneous angle, ϕ, and workpiece deformation, $\delta_{m,j}$. In the 2D cutting model, the cutting thickness is divided into two parts, $h_{j}^{1}$ and $h_{j}^{2}$, and the current position is judged by the intersection of *P* and *Q* (*P* is the intersection of the micro-cutting unit and the unprocessed surface, and *Q* is the intersection of the micro-cutting unit and the machined surface), as shown in Figure 3.

(12) $$\begin{array}{cc} h_{j}^{1}(\phi )=R-\frac{\left(R-a_{e})+\delta_{mj}(\phi \right)}{\tan\left(\phi \right)}\sqrt{\tan^{2}(\phi )+1} & \left(1\leq j\leq n\right) \end{array}$$

(13) $$h_{j}^{2}(\phi )=R-\sqrt{(R^{2}+f^{2}+\delta_{mj}^{2})+2{\left(f\cos\phi -\delta_{mj}\sin\phi \right)}^{2}+2(f\cos\phi -\delta_{mj}\sin\phi )\sqrt{R^{2}-{\left(f\sin\phi +\delta_{mj}\cos\phi \right)}^{2}}}$$

where $\phi =\begin{array}{cc} \phi_{st}+(n-j)d\phi & \left(1\leq j\leq n\right) \end{array}$.

The expression of discriminant points *P* and *Q* is:

(14) $$P=\begin{array}{cc} \frac{\left(R-a_{e})+\delta_{mj}(\phi \right)}{\tan(\phi )} & \left(P\geq R\cdot \cos(\phi_{st})-f\right) \end{array}$$

(15) $$Q=\begin{array}{cc} \cos\phi \left[\left(\delta_{mj}\sin\phi -f\right)+\sqrt{R^{2}-{\left(f\sin\phi +\delta_{mj}\cos\phi \right)}^{2}}\right] & \left(Q<R\cdot \cos(\phi_{st})-f\right) \end{array}$$

when the *Q* point disappears in the cutting process, the micro-cutting unit moves out of the workpiece, the last *Q* point is the exit angle and the *Y* direction coordinates of the *Q* point are the residual height.

Through analyzing the contact relationship between the tool and the workpiece, the coupling relationship between milling force, *Fy*, instantaneous cutting thickness and workpiece deformation is calculated iteratively. The initial conditions need to be set before the calculation begins: When the first cutting micro-element is rotated to $\left(\phi_{en}^{}+d\phi \right)$, it is assumed that the workpiece has no deflection deformation, therefore $\delta_{m}=0$. At this time, $h_{i,j}\left(\phi_{en}+d\phi \right)$ (j=1) is obtained by Equation (12), and the milling force $F_{s,i,j}\left(\phi_{en}+d\phi \right)$ (j=1) can be obtained by bringing it into Equation (4). The milling force $F_{s,i,j}\left(\phi_{en}+d\phi \right)$ (j=1) generated by the tool acting on the workpiece is brought into Equation (11) to calculate the workpiece deformation $\delta_{m,j}$ (j=1). The deformation $\delta_{m,j}$ (j=1) is taken as a known condition into the deformation calculation of the (*j* + 1)th cutting disk in the next layer. The matrix expression about $\delta_{m,j}$ is obtained by the above iterative calculation as:

(16) $$\delta_{m,j}=\left[\begin{array}{ccccccc} \delta_{m,1}\left(\phi_{1}\right) & 0 & 0 & \cdots & 0 & 0 & 0\\ \delta_{m,1}\left(\phi_{2}\right) & \delta_{m,2}\left(\phi_{1}\right) & 0 & \cdots & 0 & 0 & 0\\ \delta_{m,1}\left(\phi_{3}\right) & \delta_{m,2}\left(\phi_{2}\right) & \delta_{m,3}\left(\phi_{1}\right) & \cdots & 0 & 0 & 0\\ \vdots & \vdots & \vdots & \ddots & \vdots & \vdots & \vdots \\ \delta_{m,1}\left(\phi_{n-2}\right) & \delta_{m,2}\left(\phi_{n-3}\right) & \delta_{m,3}\left(\phi_{n-4}\right) & \cdots & \delta_{m,(j-2)}\left(\phi_{1}\right) & 0 & 0\\ \delta_{m,1}\left(\phi_{n-1}\right) & \delta_{m,2}\left(\phi_{n-2}\right) & \delta_{m,3}\left(\phi_{n-3}\right) & \cdots & \delta_{m,(j-2)}\left(\phi_{2}\right) & \delta_{m,(j-1)}\left(\phi_{1}\right) & 0\\ \delta_{m,1}\left(\phi_{n}\right) & \delta_{m,2}\left(\phi_{n-1}\right) & \delta_{m,3}\left(\phi_{n-2}\right) & \cdots & \delta_{m,(j-2)}\left(\phi_{3}\right) & \delta_{m,(j-1)}\left(\phi_{2}\right) & \delta_{m,j}\left(\phi_{1}\right)\\ 0 & \delta_{m,2}\left(\phi_{n}\right) & \delta_{m,3}\left(\phi_{n-1}\right) & \cdots & \delta_{m,(j-2)}\left(\phi_{4}\right) & \delta_{m,(j-1)}\left(\phi_{3}\right) & \delta_{m,j}\left(\phi_{2}\right)\\ 0 & 0 & \delta_{m,3}\left(\phi_{n}\right) & \cdots & \delta_{m,(j-2)}\left(\phi_{5}\right) & \delta_{m,(j-1)}\left(\phi_{4}\right) & \delta_{m,j}\left(\phi_{3}\right)\\ \vdots & \vdots & \vdots & \ddots & \vdots & \vdots & \vdots \\ 0 & 0 & 0 & \cdots & \delta_{m,(j-2)}\left(\phi_{n}\right) & \delta_{m,(j-1)}\left(\phi_{n-1}\right) & \delta_{m,j}\left(\phi_{n-2}\right)\\ 0 & 0 & 0 & \cdots & 0 & \delta_{m,(j-1)}\left(\phi_{n}\right) & \delta_{m,j}\left(\phi_{n-1}\right)\\ 0 & 0 & 0 & \cdots & 0 & 0 & \delta_{m,j}\left(\phi_{n}\right) \end{array}\right]$$

## 3. Definition of Surface Topography

In the milling process of thin-walled parts, the cutting force is the main factor affecting workpiece quality. The cutting force induces the deformation of thin-walled parts and changes the tool–workpiece contact relationship, which will affect the geometric accuracy and surface topography of the workpiece.

### 3.1. Machining Surface Forming

In the side milling process, due to the change of cutting force, the contact relationship caused by the static deflection of the tool–workpiece is not constant along the axial cutting depth. According to the cutting parameters, the tool geometry presents different surface errors and surface roughness [37,38]. Therefore, the accurate establishment of the tool–workpiece contact relationship is the key factor to predict milling force.

Different tool–workpiece contact relationships present different cutting force signal shapes [39,40]. The effects of cutting parameters ($a_{e}$, $a_{p}$) and tool geometry parameters (*D*, β) on $F_{Y}(\phi )$ are defined. The radial angle, $\alpha_{en}$, is calculated as $a_{e}$:

(17) $$\alpha_{en}=\phi_{ex}-\phi_{st}=a\cos\left(1-2\cdot a_{e}/D\right)$$

The formula of the axial meshing angle, $\alpha_{sw}$, is defined by $a_{p}$:

(18) $$\alpha_{sw}=2\cdot a_{p}\cdot \tan\left(\beta \right)/D$$

The key angles of tool–workpiece contact are defined by the radial meshing angle, $\alpha_{en}$, and axial meshing angle, $\alpha_{sw}$:

(19) $$\theta_{1}=\phi_{st}$$

(20) $$\theta_{2}=\phi_{st}+\alpha_{en}$$

(21) $$\theta_{3}=\phi_{st}+\alpha_{sw}$$

(22) $$\theta_{4}=\phi_{st}+\alpha_{sw}+\alpha_{en}$$

**Figure 4:**
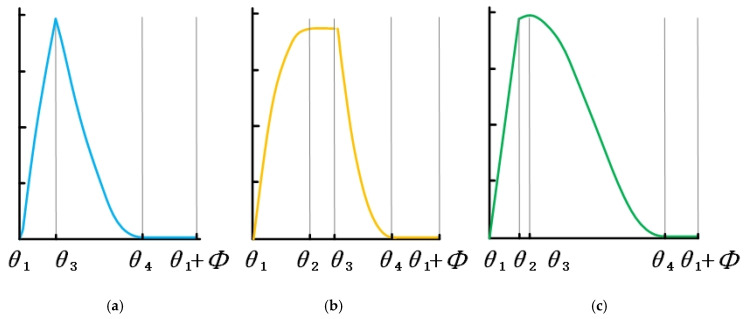
The waveform of the force signal for a single flute in a period. (**a**) Type I, (**b**) Type II and (**c**) Type III [40].

$\theta_{1}$ and $\theta_{2}$ represent the entry angle, $\phi_{st}$, and the exit angle, $\phi_{ex}$, at the bottom of $a_{p}$, respectively. Similarly, $\theta_{3}$ and $\theta_{4}$ represent the entry angle, $\phi_{st}$, and the exit angle, $\phi_{ex}$, at the top of $a_{p}$, respectively. The values of $\theta_{1}$, $\theta_{2}$, $\theta_{3}$ and $\theta_{4}$ depend on cutting parameters ($a_{e}$,$a_{p}$) and tool geometric parameters (β, *D*), which can describe the shape of the cutting force, $F_{Y}(\phi )$. The value of $\alpha_{en}$ and $\alpha_{sw}$ corresponding to cutting parameters ($a_{e}$,$a_{p}$) will have an obvious influence on $F_{Y}(\phi )$. According to the comprehensive comparison of $\alpha_{en}$, $\alpha_{sw}$ and the corresponding key angle $\theta_{m}\left(m=1,2,3,4\right)$, three shapes of the $F_{Y}(\phi )$ curve can be determined [40], as shown in Figure 4.

**Figure 5:**
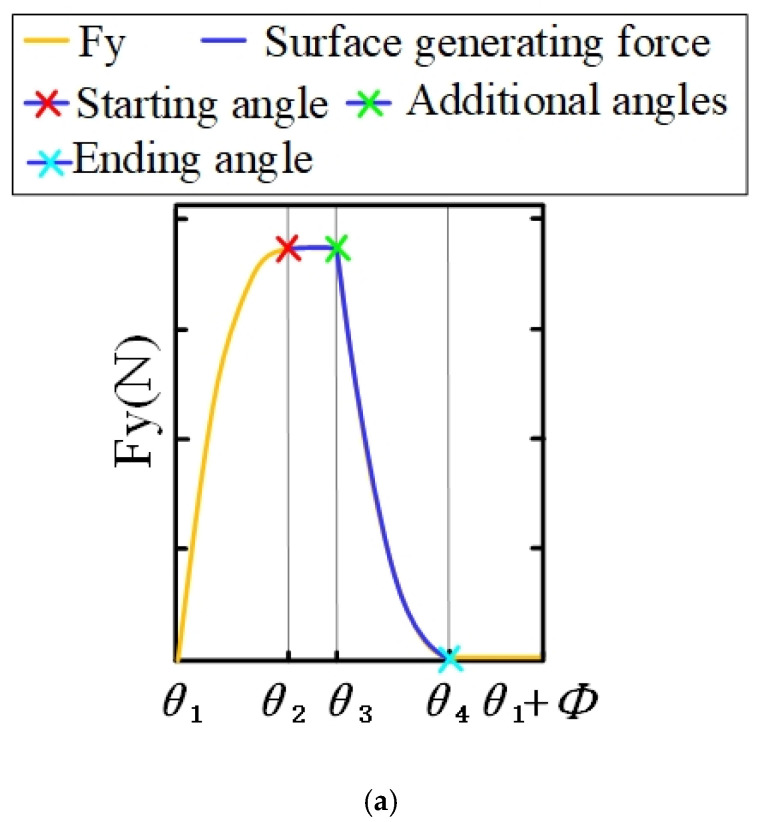

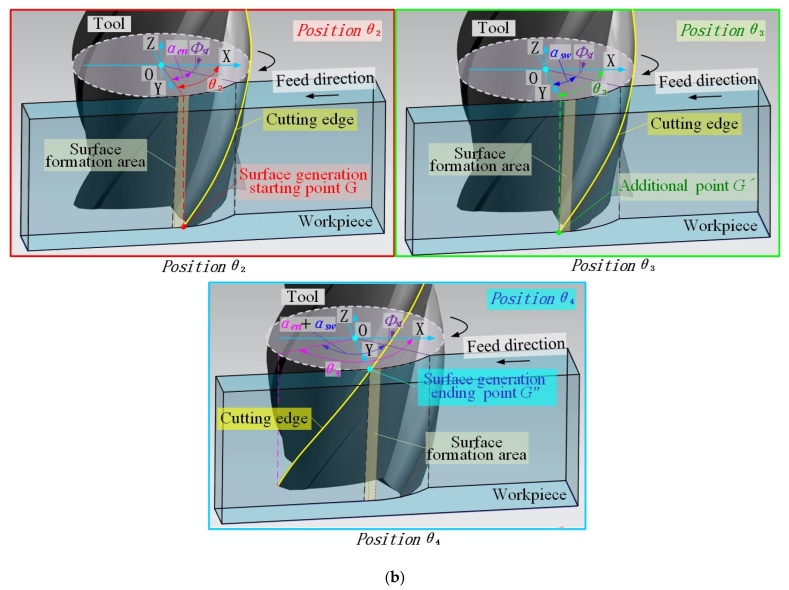
The key angle of surface generation. (**a**) The key angle selection of cutting force. (**b**) Display of key angles. The contact relationship of the tool–workpiece affects the waveform of the force signal, which also affects the surface profile of the workpiece. This correlation is applicable to any side milling process.

In the formation of the machined surface, the key angle of tool rotation and the cutting force, $F_{Y}(\phi )$, are used to describe the area of surface formation. The boundary in the surface-forming process is determined by the key angles $\theta_{2}$ (surface-forming starting angle) and $\theta_{4}$ (surface-forming ending angle), as shown in Figure 5.

### 3.2. Surface Topography Model

In the process of peripheral milling, the machined surface is the working surface left by the tool to remove the redundant material of the workpiece. The motion trajectories of different cutting edges of the end mill are trochoid curves, which produce the surface topography of grooves on the milling surface.

**Figure 6:**
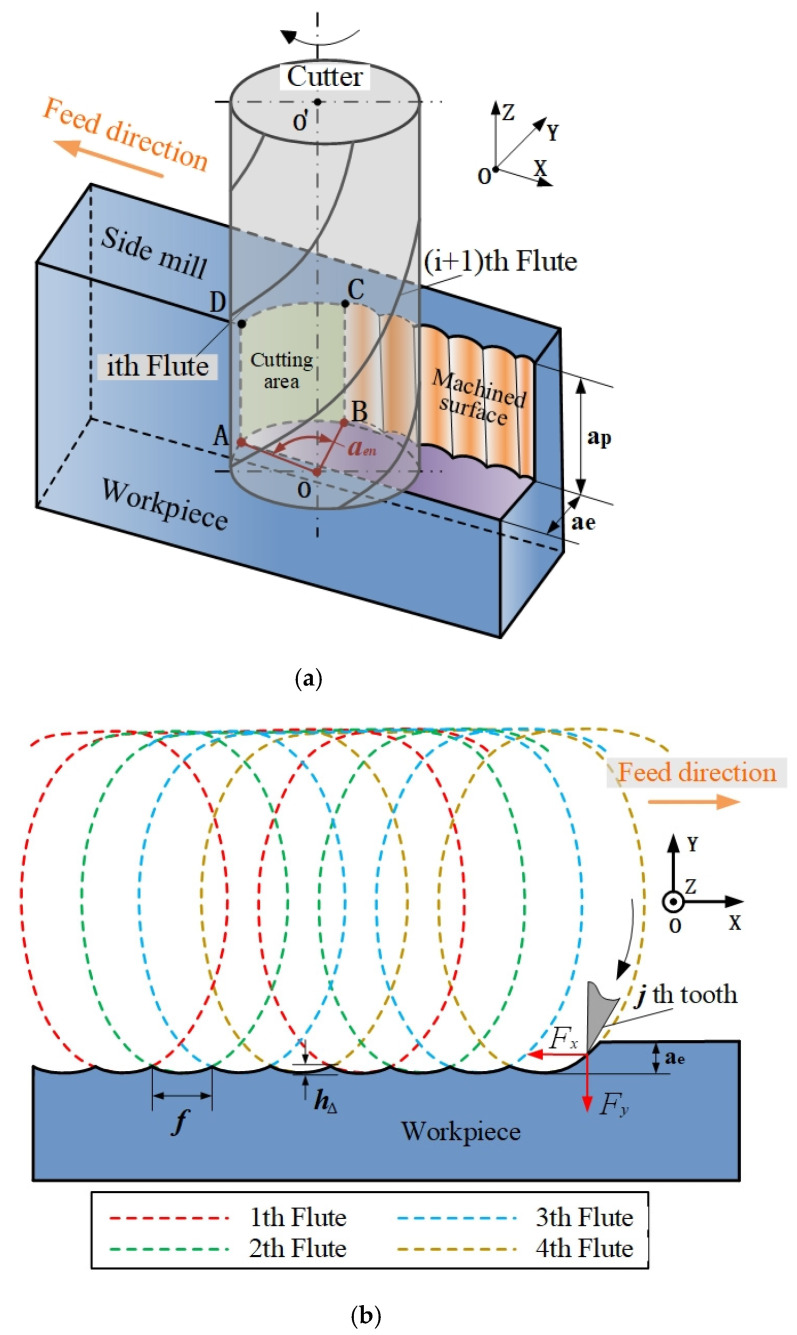
The formation of the peripheral milling surface. (**a**) The formation of the side milling surface. (**b**) 2D trajectory of the tool tooth.

Without considering vibration, deflection and other issues, the grooves on the workpiece surface are composed of cycloid arcs with the same length. The distance of each groove is equal to the length of feed, fz, per tooth. In Figure 6, the tool riding process will form a machined surface. In the peripheral milling process, the side face of the workpiece is the most attractive, and the tool is in rotation and feed motion.

Figure 6 shows the coordinate system of the workpiece in the milling process, and the required trajectory for analysis depends on the geometric shape of the milling tool. *X* is the feed direction, *Y* is the feed normal direction and *Z* is the spindle direction. In the rotational motion, it can be seen that each disk is distributed along the spiral edge.

The tool has translational and harmonic motion along the *X* and *Y* directions [41], and the trajectory of the *i*th cutting edge is calculated as follows:

(23) $$\left[\begin{array}{c} X_{i,j}\\ Y_{i,j}\\ Z_{i,j} \end{array}\right]=\left[\begin{array}{c} f\cdot t+\frac{D}{2}\sin\left(S\cdot t-\frac{2\pi \cdot i}{n}\right)\\ \frac{D}{2}\cos\left(S\cdot t-\frac{2\pi \cdot i}{n}\right)\\ Z_{i,j} \end{array}\right]$$

where *D* is the tool diameter, *f* is the feed rate, *t* is the milling processing time, *S* is the spindle speed and *n* is the flute number of the tool.

In this paper, the surface topography of the sidewall in the milling process is taken as the research objective. The rotary cutting edge is along the helix angle, *β,* around the tool cylinder. According to Equation (22), the theoretical cutting path in the *Z* direction of the *i*th cutting edge is shown as follows:

(24) $$\left[\begin{array}{c} X_{i,j}\\ Y_{i,j}\\ Z_{i,j} \end{array}\right]=\left[\begin{array}{l} f\cdot t+\frac{D}{2}\sin\left(S\cdot t-\frac{2\pi \cdot i}{n}-\frac{2\cdot Z_{i,j}\tan\left(\beta \right)}{D}\right)\\ \frac{D}{2}\cos\left(S\cdot t-\frac{2\pi \cdot i}{n}-\frac{2\cdot Z_{i,j}\tan\left(\beta \right)}{D}\right)\\ Z_{i,j} \end{array}\right]$$

### 3.3. Define the Surface Generation Area

In the milling process, most of the time is spent in the material removal process, and only a small part of the time is used to form the surface contour. From the cut-in to cut-out process, the machined surface is formed when the flute rotates to the specified angle. It is an important factor to accurately define the forming range of the machined surface in the numerical calculation of surface topography and roughness.

**Figure 7:**
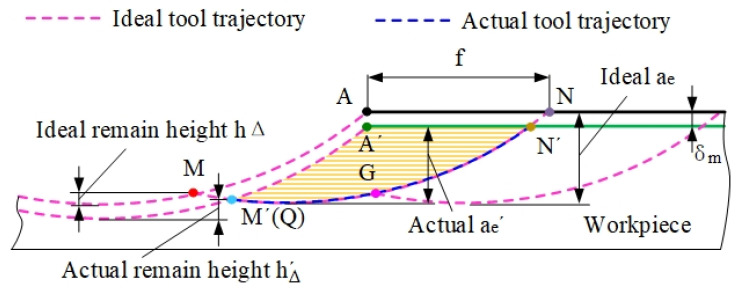
Surface topography formation area [38].

In the side-milling surface generation process, there are residual materials on the surface, and the residual material height and arc contour form the machined surface. The distance between adjacent residual heights is equal to the feed per tooth, *f*, as shown in Figure 7. When the tool rotates to $\theta_{2}$, the first disk enters the surface generation area, where the tooltip of the first disk intersects with the line $L_{f}$ at $G_{1}$. As the tool rotates the *j*th disk in the sequence into the workpiece, the surface topography of the current layer begins to form when the intersection $G_{1}$ between the *j*th disk and line $L_{f}$ appears. The $G_{j}$ coordinate is expressed as:

(25) $$G_{j}=\left[\begin{array}{c} X_{j}\left(\phi \right)\\ Y_{j}\left(\phi \right) \end{array}\right]=\left[\begin{array}{c} f/2\\ \sqrt{R^{2}-{\left(f/2\right)}^{2}} \end{array}\right]$$

In the milling process of thin-walled parts, the actual surface trajectory deviates from the theoretical trajectory due to the deformation of the workpiece. This is because point $Q_{j}$ leaves the workpiece in advance, so the residual height of the workpiece surface is higher than the theoretical residual height. The $G_{j}$ point is the starting point in the surface generation process. With the rotation of the tool, the cutting process is completed, and the machined surface is formed when $G_{j}$ and $Q_{j}$ coincide.

## 4. The Surface Topography Simulation

### 4.1. Surface Topography Simulation Model

After obtaining the tool geometry parameters and cutting parameters, the corresponding coordinates of the ideal machined surface topography can be calculated. Due to the flexible deformation of the workpiece, the ideal cutting trajectory remaining on the workpiece surface deviates, which affects the machined surface topography. In order to improve the surface quality of thin-walled parts, the coupling relationship model of force and deformation is established, as well as the prediction model of milling surface topography for thin-walled parts.

The actual trajectory model after deformation is as follows:

(26) $$\left[\begin{array}{c} X_{i,j}\\ Y_{i,j}\\ Z_{i,j} \end{array}\right]=\left[\begin{array}{l} f\cdot t+\frac{D}{2}\sin\left(S\cdot t-\frac{2\pi \cdot i}{n}-\frac{2\cdot Z_{i,j}\tan\left(\beta \right)}{D}\right)\\ \frac{D}{2}\cos\left(S\cdot t-\frac{2\pi \cdot i}{n}-\frac{2\cdot Z_{i,j}\tan\left(\beta \right)}{D}\right)\\ Z_{i,j} \end{array}\right]+\left[\begin{array}{c} \delta_{X}\\ \delta_{Y}\\ 0 \end{array}\right]$$

where $\delta_{X}$ and $\delta_{Y}$ are the deformation under the force action.

**Figure 8:**
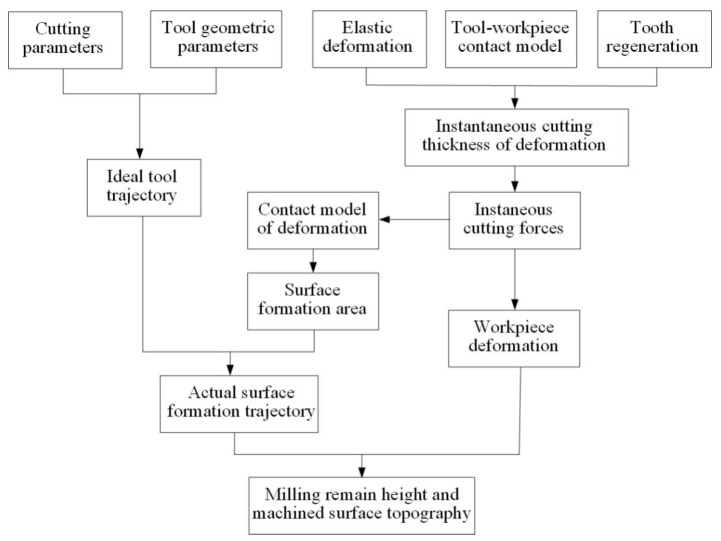
Milled surface topography model.

By integrating the mechanical model of the milling process, the flexible deformation model and the tool–workpiece contact model, the side-milling surface topography model is established. The flowchart is shown in Figure 8.

The tool geometry and cutting parameters are input to calculate the instantaneous cutting thickness, cutting force and deformation. The deformation displacement of the workpiece under the current force action is obtained through the force–deformation coupling model. The actual milling trajectory is obtained by fusing the deformation into the ideal trajectory.

### 4.2. Simulation Model

**Figure 9:**
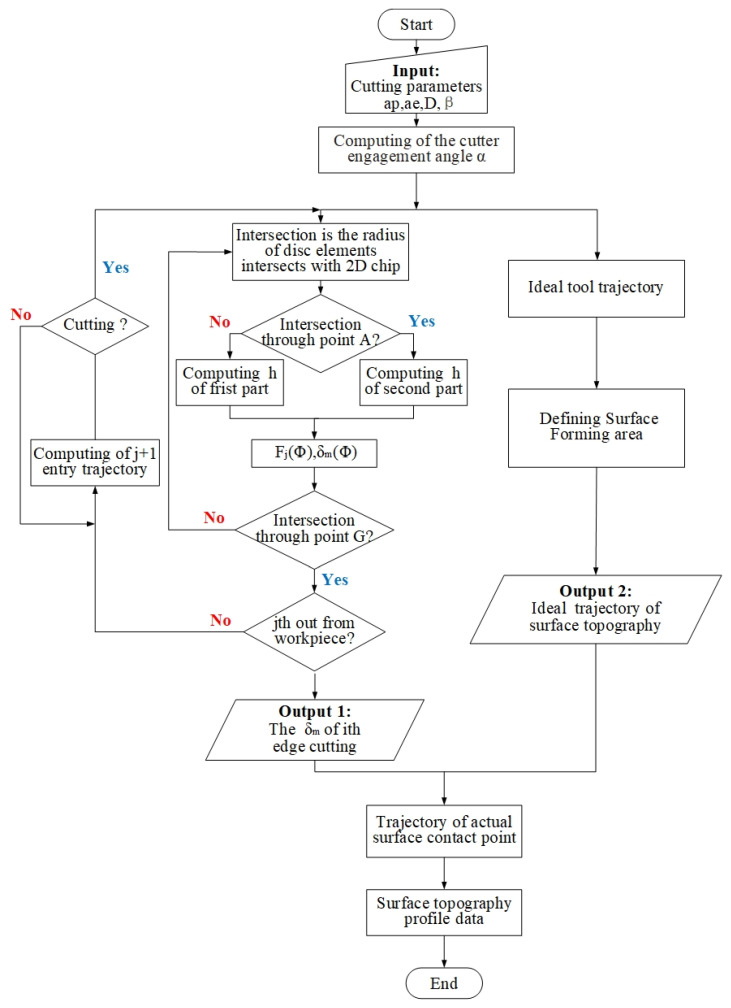
The flowchart of milling surface topography simulation of thin-walled parts.

In the milling process of thin-walled parts, the surface topography is predicted by the superposition of the workpiece surface profile and the deformation calculated by the cutting force. Figure 9 describes the calculation flowchart of milling topography for thin-walled parts. In the simulation process, the actual cutting trajectory is obtained through the tool–workpiece contact relationship after deformation, and the surface topography is predicted.

Due to the end milling tool having spiral edges, the instantaneous cutting thickness and contact angle change at different axial heights. The tool is dispersed in the axial direction to simulate the actual cutting process. According to the judgment points *P* and *Q*, the instantaneous cutting thickness of each axial disk is calculated, and the instantaneous tool–workpiece contact position is defined. The cutting force and workpiece deformation at the current moment are calculated by instantaneous cutting thickness. The trajectory points after the current deformation are recorded, and the G point is used to determine whether the current cutter tooth enters the surface-forming area. The deformation and ideal trajectory are combined to obtain the actual surface contact point.

According to the cycle path in the above process, the milling process is simulated to obtain the three-dimensional coordinates of the actual cutting trajectory of each disk at each time in the milling process of thin-walled parts. Finally, the data points of the surface topography are obtained by Boolean operation for the trajectory of the axial disk and the workpiece surface. The surface topography prediction model established in this paper reduces the cost of the calculation process and improves the calculation efficiency under the premise of ensuring the calculation accuracy.

## 5. Experimental Validation

The surface topography prediction model is verified by milling experiments of titanium alloy TC4 thin-walled parts, which is a typical material in the aerospace field. The surface roughness is measured under different cutting parameters, and the accuracy of the prediction model is verified by experimental results.

### 5.1. Set-Up

**Figure 10:**
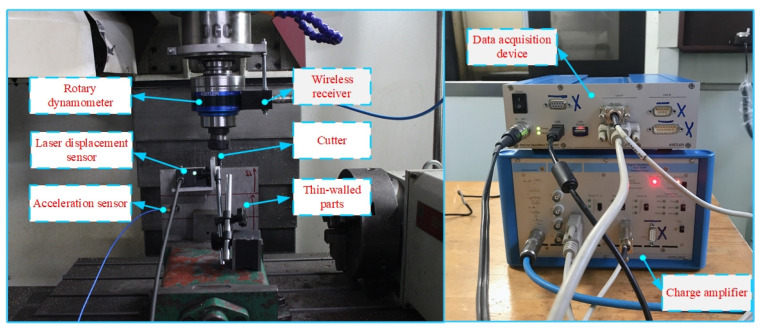
Photograph of the milling system.

Experiments are carried out on a three-axis CNC machine tool (Figure 10), and the model is VDL-1000E. The spindle speed range is 40–8000 r/min, the cutting speed range is 1–10,000 mm/min and the repeat positioning accuracy is *X*: 0.007 mm, *Y* and *Z*: 0.006 mm. In the cutting process, the rotary dynamometer (Kistler 5236B) and charge amplifier (5238B) are used to acquire the cutting force signal in the milling process. The displacement sensor (KEYENCE LKH020) is used to collect the deformation of thin-walled parts. The white light interferometer (CCI Map Taylor Hobson, Leicester, UK) is used to measure the surface roughness of the workpiece. The tool is a cemented carbide end milling cutter with a diameter of 10 mm, and the number of cutting edges is 4.

The material of the test specimens is TC4 titanium alloy and the elastic modulus, E, is 110 GPa. The geometric size of the workpiece is 200 × 200 × 8 mm, the fixture clamps the workpiece and the extension length is 150 mm. Milling experiments are carried out with a hard alloy flat-end tool with a 10 mm diameter and 4 teeth (Zhuzhou Cemented Carbide Cutting Tool Company, Hunan, China, solid carbide end mills, model is VSM-4E-D10.0).

### 5.2. Experimental Validation

**Figure 11:**
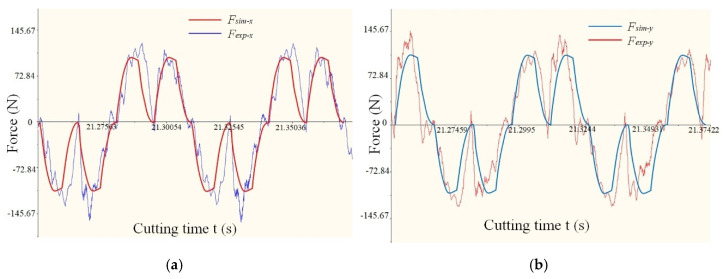

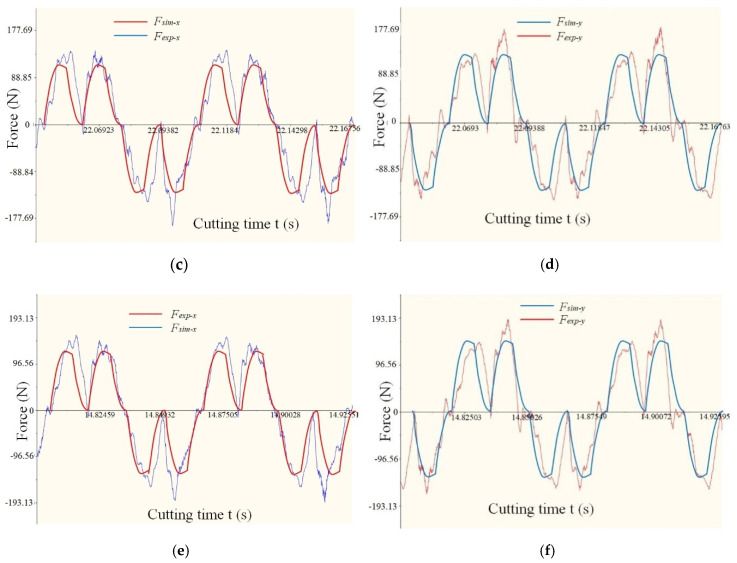
Experimental and simulation comparison of milling force. (**a**) The 1st set of X-Force. (**b**) The 1st set of X-Force. (**c**) The 4th set of X-Force. (**d**) The 4th set of X-Force. (**e**) The 6th set of X-Force. (**f**) The 6th set of X-Force.

In the milling process of thin-walled parts, the milling force is the main factor to induce workpiece deformation, part accuracy deviation and poor surface quality. The milling force verification experiment is carried out using the experimental device in Section 5.1 to verify the effectiveness of the established milling force prediction model. The milling force experiment is carried out by the milling parameters in Table 1, and the simulation and experimental results of the milling force X and Y directions in Figure 11 are obtained.

It can be seen from the results in Figure 11 that when the workpiece deformation is introduced into the calculation of milling force, the maximum relative error of milling force is 8.49–17.32%, and the error range is acceptable. The main factors that cause the deviation between the simulation value and the experimental measurement value are: the forced vibration of the cutting process produced by the cutting system, such as the centrifugal force produced by the rotary motion of the spindle and the tool, the periodic change of the cutting force caused by the inhomogeneity of the cutting process itself and the inertial impact force produced by the moving parts will directly affect the cutting force. The main cutting force causing workpiece deformation in the model is *Fy*, and the influence of *Fx* on deformation is ignored. However, the consistency between the predicted value and the experimental value is good, and the error range is acceptable, which verifies the effectiveness of the milling force prediction model. Milling force and workpiece deformation need to be measured at the same time. The measured deformation value is compared with the predicted value, and the maximum deflection deformation value is used as the evaluation index in the comparison process, as shown in Table 1.

The surface topography and surface roughness, Ra, of titanium alloy thin-walled parts under different milling parameters are measured to verify the effectiveness of the model. The elastic modulus, *E*, of the workpiece is 110 GPa. In order to improve the reliability of the measurement results, six measurement positions are selected for each machined surface, and each measurement point is implemented three times. The average value of the measured results, Ra, is taken as the experimental result for comparison. Six groups of experimental results are compared with simulation results, as shown in Table 2.

From the results of Table 1, the accuracy of the prediction model is verified by measured results. The results’ consistency is good in the range of used parameters. The maximum relative error between simulated and measured surface roughness is 13.09%, and the average relative error is 7.45%. There are some differences among the results, which may be caused by other factors (tool wear, cutting vibration and other factors).

**Figure 12:**
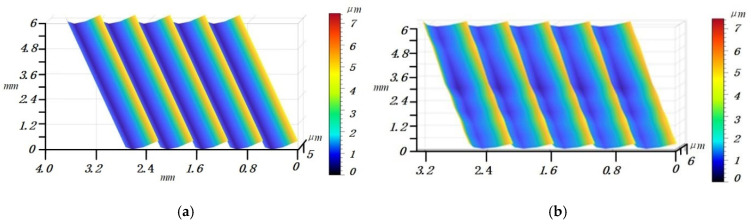
Comparison of simulation results (*a_e_* = 0.4, *a_p_* = 6 mm, *f* = 0.08 mm, *S* = 1000 rpm). (**a**) The simulation surface topography of the ideal tool path. (**b**) The first set of parameter simulation results.

Figure 12a shows the machined surface generated by the ideal tool path without considering other factors, such as deformation, wear and vibration. Figure 12b shows the first set (*a_e_* = 0.4, *a_p_* = 6 mm, *f* = 0.08 mm, *S* = 1000 rpm) of parameter simulation results, and it can be seen that the tool cutting-out trajectory has obvious deviation and the surface residual height changes.

**Figure 13:**
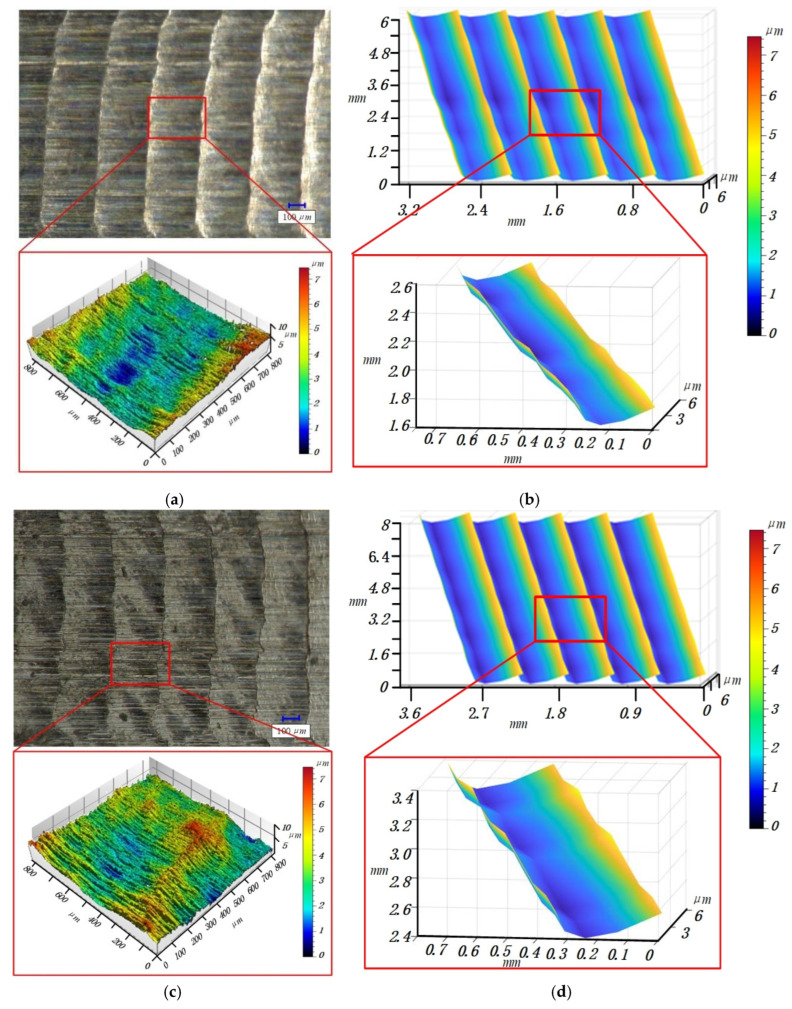

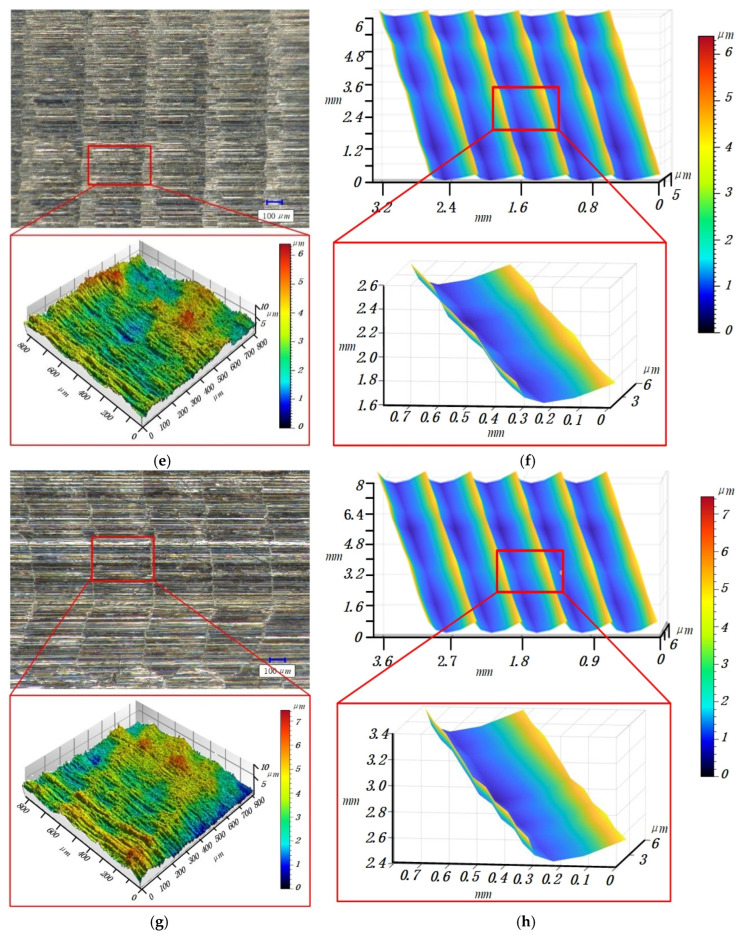
Measured (**a**,**c**,**e**,**g**) and simulated (**b**,**d**,**f**,**h**) surface topographies in Table 2. (**a**) The 1st set of measurement topography. (**b**) The 1st set of simulation topography. (**c**) The 2nd set of measurement topography. (**d**) The 2nd set of simulation topography. (**e**) The 3rd set of measurement topography. (**f**) The 3rd set of simulation topography. (**g**) The 4th set of measurement topography. (**h**) The 4th set of simulation topography.

A white light interferometer and super depth of field microscope are used to measure the three-dimensional surface profile. Figure 13a,c,e,g show the measured results of the three-dimensional surface topography of titanium alloy thin-walled parts, with the No. 1, 2, 3 and 4 milling parameters in Table 2, respectively. Figure 13b,d,f,h show the simulation results of the three-dimensional surface topography of titanium alloy thin-walled parts with the No. 1, 2, 3 and 4 milling parameters in Table 2, respectively. In the comparison of measurement and simulation images, it can be seen that the maximum residual height of the parts is basically the same. It can be seen from Figure 13a,c,e,g that the deformation causes the change of the contact relationship between the tool and the workpiece, which makes the tool deviate from the ideal cutting-out position when it moves out of the workpiece, and at the same time makes the topography of the cutting-out position present a state of fluctuation. Therefore, the established surface topography prediction model can well-predict the surface topography of the workpiece.

In Figure 13, the peak values of Figure 13a,b are 6.873 and 5.276 μm, respectively, and the error is 1.597 μm, the peak values of Figure 13c,d are 7.014 and 5.963 μm, respectively, and the difference is 1.051 μm, the peak values of Figure 13e,f are 6.007 and 4.714 μm, respectively, and the difference is 1.293 μm, and the peak values of Figure 13g,h are 6.656 and 5.317 μm, respectively, and the difference is 1.339 μm. Since titanium alloy is a difficult to process material, its processing will lead to small residues attached to the workpiece surface, resulting in sudden changes in the measured values. Therefore, Ra is used as the evaluation object in the evaluation process.

Due to the small measurement range of the white light interferometer, the deviation phenomenon when the tool cuts out the workpiece cannot be fully described. Therefore, the super depth of field microscope is used to observe the surface topography of the workpiece. In Figure 13a,g, there are obvious strip traces, which may be caused by the small built-up edge bonded on the cutting edge at this position or the micro-wear of the flank face.

**Figure 14:**
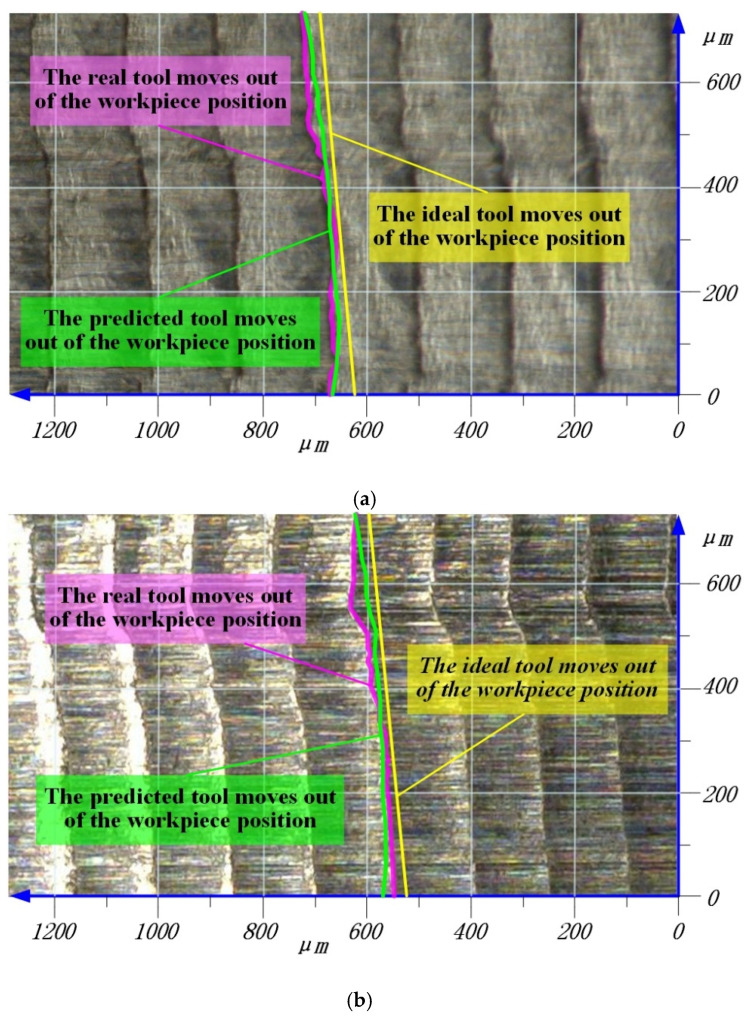
The tool moves out of the workpiece position in Table 2. (**a**) The 5th set of tools move out of the workpiece position. (**b**) The 6th set of tools move out of the workpiece position.

In the ideal milling process, the trajectory formed when the tool moves out of the workpiece in the *z*-axis direction is linear. The formation of a straight line is the set of points, M, that each disk moves out of the workpiece position. In the milling process of thin-walled parts, deformation causes the change of the tool–workpiece contact relationship. It can be seen from Figure 7 that the disk moves out of the workpiece in advance, so that the actual position of the tool moving out of the workpiece deviates from the ideal position. Figure 14 shows a comparison of the actual and simulated tool moving out of the workpiece of the 5th and 6th sets of cutting parameters in Table 2.

It is obvious from Figure 14 that the traces left by the tool on the workpiece surface show a nonlinear shape, which is caused by the deformation that makes the actual cutting trajectory deviate from the ideal trajectory. This phenomenon is consistent with the prediction model established in this paper that changes the contact relationship via deformation. In the dry cutting process, the tool flank will attach small residues. These residues will change the residual height of the workpiece surface and randomly form unpredictable surface mutations, which can be seen in Figure 14. However, the occurrence of this situation is random, which does not affect the prediction ability of the model. By comparing the simulation results with the measurement results, it can be seen that there is a good similarity.

## 6. Conclusions

In this paper, a prediction model of surface topography considering cutting force and deformation for side milling titanium alloy thin-walled parts was established. Based on the contact relationship after cutting deformation, a new surface landform formation area was calculated. Finally, the simulation results of the workpiece surface topography were in good agreement with the measured results. The research contents of this paper are as follows:

(1) Through the cutting force and the beam deformation model, the coupling calculation relationship between force and deformation was established, which can calculate the instantaneous deformation value of the workpiece (deformation value matrix). The instantaneous cutting thickness after deformation was obtained, and the contact relationship between the deformed tool and the workpiece was revealed, which changed the residual height of the machined workpiece surface. The experimental results showed that the error between the milling force prediction model and the measured value was 8.49–17.32%, and the error between the predicted deformation value and the measured deformation value was 7.45%.
(2) The cutting force was calculated according to the tool geometric and cutting parameters. By obtaining the waveform of the force signal, the range of the surface contour generation area and the key angles in the surface generation process were defined as $\theta_{2}$, $\theta_{3}$ and $\theta_{4}$. A new surface formation zone after deformation can be determined by key angles and the deformation value. According to the tool trajectory, the starting point *G* in the surface formation process was given, which provided judgment conditions for the simulation calculation.
(3) The deformation was introduced into the ideal trajectory, and the deformed tool trajectory was converted to the workpiece surface to form the final surface topography. Through the established three-dimensional surface topography simulation algorithm during the milling of thin-walled parts, the surface roughness, Ra, was obtained as the evaluation index, and the experimental results were compared. The maximum relative error of surface roughness, Ra, was 13.09%, and the average error was 7.45%. The simulation results of surface topography had good similarity with the measured results. This paper provides a reference for the prediction of surface topography and the study of milling mechanisms in side milling of thin-walled parts.

## Figures and Tables

**Table 1:** Comparison of simulation and experimental results of deformation.

No.	Radial Cutting Depth, *a_e_*/mm	Axial Cutting Depth, *a_p_*/mm	Feed Per Tooth, *f*/mm	Spindle Speed, *S*/rpm	Prediction Results (μm)	Measuring Results (μm)	Error (%)
1	0.4	6	0.08	1000	19.1	20.1	4.9
2	0.5	8	0.08	1000	19.8	21.3	7
3	0.6	6	0.08	1000	20.7	24.8	16.5

**Table 2:** Comparison of simulation and experimental results of surface roughness.

No.	Radial Cutting Depth, *a_e_*/mm	Axial Cutting Depth, *a_p_*/mm	Feed Per Tooth, *f*/mm	Spindle Speed, *S*/rpm	Prediction Results, Ra (μm)	Measuring Results, Ra (μm)	Error (%)
1	0.4	6	0.08	1000	0.3353	0.3792	13.09%
2	0.4	8	0.08	1000	0.4381	0.4602	5.04%
3	0.5	6	0.08	1000	0.3143	0.2902	−7.67%
4	0.5	8	0.08	1000	0.3358	0.3765	12.12%
5	0.6	6	0.08	1000	0.4812	0.5257	9.25%
6	0.6	8	0.08	1000	0.3627	0.4093	12.85%

## Data Availability

Not applicable.

## References

[1] Caixu Y., Juntao Z., Xianli L., Zhitao C., Yuesheng L., Lihui W. (2021). Research progress on machining deformation of thin-walled parts in milling process. Acta Aeronaut. Astronaut. Sin..

[2] Fei J., Lin B., Yan S., Ding M., Zhang J., Ji C., Zhang X. (2018). Modeling of surface roughness for manufactured thin-walled structure. Proc. Inst. Mech. Eng. Part B J. Eng. Manuf..

[3] Lee K.Y., Kang M.C., Jeong Y.H., Lee D.W., Kim J.S. (2001). Simulation of surface roughness and profile in high-speed end milling. J. Mater. Process. Technol..

[4] Omar O., El-Wardany T., Ng E., Elbestawi M.A. (2007). An improved cutting force and surface topography prediction model in end milling. Int. J. Mach. Tools Manuf..

[5] Zhang X., Yu T., Zhao J. (2020). Surface generation modeling of micro milling process with stochastic tool wear. Int. J. Precision Eng..

[6] Yang D., Liu Z. (2015). Surface plastic deformation and surface topography prediction in peripheral milling with variable pitch end mill. Int. J. Mach. Tools Manuf..

[7] Arizmendi M., Campa F.J., Fernández J., de Lacalle L.L., Gil A., Bilbao E., Lamikiz A. (2009). Model for surface topography prediction in peripheral milling considering tool vibration. CIRP Ann. Manuf. Technol..

[8] Arizmendi M., Jiménez A. (2019). Modelling and analysis of surface topography generated in face milling operations. Int. J. Mech. Sci..

[9] Torta M., Albertelli P., Monno M. (2020). Surface morphology prediction model for milling operations. Int. J. Adv. Manuf. Technol..

[10] Montgomery D., Altintas Y. (1991). Mechanism of Cutting Force and Surface Generation in Dynamic Milling. J. Eng. Ind..

[11] Yan B., Zhu L., Liu C. (2020). Prediction model of peripheral milling surface geometry considering cutting force and vibration. Int. J. Adv. Manuf. Technol..

[12] Maruda R.W., Wojciechowski S., Szczotkarz N., Legutko S., Mia M., Gupta M.K., Krolczyk G.M. (2020). Metrological analysis of surface quality aspects in minimum quantity cooling lubrication. Measurement.

[13] Zhang G., Li J., Chen Y., Huang Y., Shao X., Li M. (2014). Prediction of surface roughness in end face milling based on Gaussian process regression and cause analysis considering tool vibration. Int. J. Adv. Manuf. Technol..

[14] Chen H.Q., Wang Q.H. (2019). Modelling and simulation of surface topography machined by peripheral milling considering tool radial runout and axial drif. Proc. Inst. Mech. Eng. Part B J. Eng. Manuf..

[15] Pimenov D.Y., Hassui A., Wojciechowski S., Mia M., Magri A., Suyama D.I., Gupta M.K. (2019). Effect of the Relative Position of the Face Milling Tool towards the Workpiece on Machined Surface Roughness and Milling Dynamics. Appl. Sci..

[16] Wojciechowski S., Wiackiewicz M., Krolczyk G.M. (2018). Study on metrological relations between instant tool displacements and surface roughness during precise ball end milling. Measurement.

[17] Ulutan D., Ozel T. (2011). Machining induced surface integrity in titanium and nickel alloys: A review. Int. J. Mach. Tools Manuf..

[18] Bolar G., Das A., Joshi S.N. (2018). Measurement and analysis of cutting force and product surface quality during end-milling of thin-wall components. Measurement.

[19] Hao Y., Liu Y. (2017). Analysis of milling surface roughness prediction for thin-walled parts with curved surface. Int. J. Adv. Manuf. Technol..

[20] Chuchala D., Dobrzynski M., Pimenov D.Y., Orlowski K.A., Krolczyk G., Giasin K. (2021). Surface Roughness Evaluation in Thin EN AW-6086-T6 Alloy Plates after Face Milling Process with Different Strategies. Materials.

[21] Kline W.A., Devor R.E., Shareef I.A. (1982). The Prediction of Surface Accuracy in End Milling. Trans. ASME J. Eng. Ind..

[22] Arizmendi M., Fernández J., Gil A., Veiga F. (2009). Effect of tool setting error on the topography of surfaces machined by peripheral milling. Int. J. Mach. Tools Manuf..

[23] Lotfi B., Zhong Z.W., Khoo L.P. (2009). Variable feed rates and variable machine forces for a constant material removal rate and constant cutting force along Pythagorean-hodograph curves. Int. J. Adv. Manuf. Technol..

[24] Xie D., Ding J., Liu F., Jiang Z., Du L., Wang W., Song Z. (2014). Modeling errors forming abnormal tool marks on a twisted ruled surface in flank milling of the five-axis CNC. J. Mech. Sci. Technol..

[25] Liu G., Dang J., Li C., Ming W., An Q., Chen M. (2019). Investigation on the vibration and machined surface quality in tilt side milling of thin-walled plates. Int. J. Adv. Manuf. Technol..

[26] Wang W., Li Q., Jiang Y. (2020). A novel 3D surface topography prediction algorithm for complex ruled surface milling and partition process optimization. Int. J. Adv. Manuf. Technol..

[27] Zhang X., Zhang J., Pang B., Zhao W. (2016). An accurate prediction method of cutting forces in 5-axis flank milling of sculptured surface. Int. J. Mach. Tools Manuf..

[28] Wang W., Kweon S.H., Yang S.H. (2005). A study on roughness of the micro-end-milled surface produced by a miniatured machine tool. J. Mater. Process. Technol..

[29] Denkena B., Köhler J., Sellmeier V., Mörke T. (2011). Topography prediction of resilient parts after flank milling with chamfered tools. Prod. Eng..

[30] Yildiz A.R. (2013). A new hybrid differential evolution algorithm for the selection of optimal machining parameters in milling operations. Appl. Soft Comput..

[31] Subramanian M., Sakthivel M., Sooryaprakash K., Sudhakaran R. (2013). Optimization of Cutting Parameters for Cutting Force in Shoulder Milling of Al7075-T6 Using Response Surface Methodology and Genetic Algorithm. Procedia Eng..

[32] Yildiz A.R. (2013). Cuckoo search algorithm for the selection of optimal machining parameters in milling operations. Int. J. Adv. Manuf. Technol..

[33] Budak E., Altintas Y. (1994). Peripheral milling conditions for improved dimensional accuracy. Int. J. Mach. Tools Manuf..

[34] Altinta Y., Lee P. (1996). A General Mechanics and Dynamics Model for Helical End Mills. CIRP Ann. Manuf. Technol..

[35] Desai K.A., Rao P. (2012). On cutter deflection surface errors in peripheral milling. J. Mater. Process. Tech..

[36] Calleja A., Bo P., González H., Bartoň M., de Lacalle L.L. (2018). Highly-accurate 5-axis flank CNC machining with conical tools. Int. J. Adv. Manuf. Technol..

[37] Chen Z., Yue C., Liang S.Y., Liu X., Li H., Li X. (2020). Iterative from error prediction for side-milling of thin-walled parts. Int. J. Adv. Manuf. Technol..

[38] Yue C., Chen Z., Liang S.Y., Gao H., Liu X. (2019). Modeling machining errors for thin-walled parts according to chip thickness. Int. J. Adv. Manuf. Technol..

[39] Dépincé P., Hascoet J.Y. (2006). Active integration of tool deflection effects in end milling. Part 2. Compensation of tool deflection. Int. J. Mach. Tools Manuf..

[40] Yang L., DeVor R.E., Kapoor S.G. (2005). Analysis of Force Shape Characteristics and Detection of Depth-of-Cut Variations in End Milling. J. Manuf. Sci. Eng..

[41] Lu X., Hu X., Jia Z., Liu M., Gao S., Qu C., Liang S.Y. (2018). Model for the prediction of 3D surface topography and surface roughness in micro-milling Inconel 718. Int. J. Adv. Manuf. Technol..

